# Striped Expression of Leucine-Rich Repeat Proteins Coordinates Cell Intercalation and Compartment Boundary Formation in the Early *Drosophila* Embryo

**DOI:** 10.3390/sym15081490

**Published:** 2023-07-27

**Authors:** Chloe A. Kuebler, Adam C. Paré

**Affiliations:** Department of Biological Sciences, University of Arkansas, Fayetteville, AR 72701, USA;

**Keywords:** convergent extension, planar polarity, Toll receptors, Tartan, Ten-m, Cirl, leucine-rich repeat, cell intercalation, compartment boundary

## Abstract

Planar polarity is a commonly observed phenomenon in which proteins display a consistent asymmetry in their subcellular localization or activity across the plane of a tissue. During animal development, planar polarity is a fundamental mechanism for coordinating the behaviors of groups of cells to achieve anisotropic tissue remodeling, growth, and organization. Therefore, a primary focus of developmental biology research has been to understand the molecular mechanisms underlying planar polarity in a variety of systems to identify conserved principles of tissue organization. In the early *Drosophila* embryo, the germband neuroectoderm epithelium rapidly doubles in length along the anterior-posterior axis through a process known as convergent extension (CE); it also becomes subdivided into tandem tissue compartments through the formation of compartment boundaries (CBs). Both processes are dependent on the planar polarity of proteins involved in cellular tension and adhesion. The enrichment of actomyosin-based tension and adherens junction-based adhesion at specific cell-cell contacts is required for coordinated cell intercalation, which drives CE, and the creation of highly stable cell-cell contacts at CBs. Recent studies have revealed a system for rapid cellular polarization triggered by the expression of leucine-rich-repeat (LRR) cell-surface proteins in striped patterns. In particular, the non-uniform expression of Toll-2, Toll-6, Toll-8, and Tartan generates local cellular asymmetries that allow cells to distinguish between cell-cell contacts oriented parallel or perpendicular to the anterior-posterior axis. In this review, we discuss (1) the biomechanical underpinnings of CE and CB formation, (2) how the initial symmetry-breaking events of anterior-posterior patterning culminate in planar polarity, and (3) recent advances in understanding the molecular mechanisms downstream of LRR receptors that lead to planar polarized tension and junctional adhesion.

## 1. Introduction

**Figure 1.**
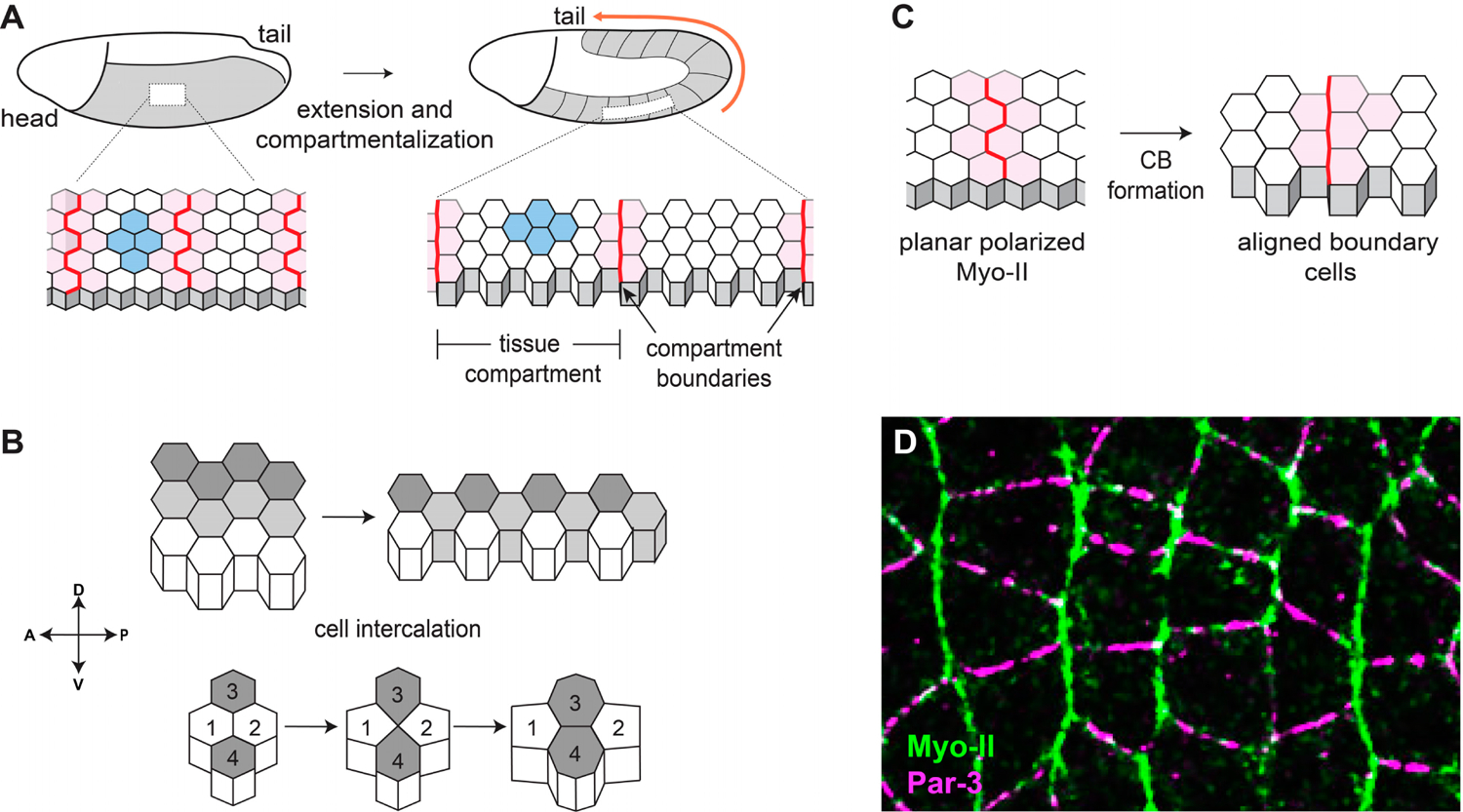
Convergent extension and compartmentalization of the early *Drosophila* embryo is mediated by planar polarity. (**A**) The germband neuroectoderm in the early *Drosophila* embryo extends along the anterior-posterior axis through CE and divides into multiple tissue compartments. (**B**) CE in the neuroectoderm is mediated by cell intercalation, which elongates the tissue along one axis while narrowing along the perpendicular axis. (**C**) Compartmentalization of the early embryo establishes non-mixing groups of cells through the formation of compartment boundaries. (**D**) At the onset of CE, contractile Myo-II (green) becomes planar polarized to AP contacts and adhesive Par-3 (magenta) is enriched at DV contacts.

During animal development, groups of cells often undergo coordinated changes in position to establish the embryonic body plan and generate complex tissues. Convergent extension (CE) is a common developmental process in which a group of cells elongates along one axis (i.e., extension) while narrowing along another (i.e., convergence) (Figure 1A) [1–4]. During CE, individual cells undergo dynamic changes in cell morphology that allow for the rearrangement of individual cells within the tissue. In epithelial tissues, CE involves rows of cells along the axis of elongation separating and mixing with cells from adjacent rows through directional cell intercalation. Cell intercalation is driven by the contraction of cell-cell contacts oriented perpendicular to the axis of elongation and the formation of new cell-cell contacts oriented parallel to the elongating axis, a process known as neighbor exchange (Figure 1B). Actin-based protrusions perpendicular to the axis of elongation also help epithelial cells to crawl between their neighbors [5]. By contrast, contacts that were originally aligned with the axis of elongation remain stable. Another important developmental process that shares similarities with CE is the formation of compartment boundaries (CBs), which are structures consisting of aligned, high-tension cell-cell contacts that divide epithelial tissues into non-mixing compartments (Figure 1C) [6–8]. Importantly, to have organized, rather than random, cell intercalation, and to correctly position CBs, there must be mechanisms for encoding axial information within the tissue that instruct cells to contract specific subsets of contacts while stabilizing others. Elucidating the molecular basis of cell intercalation and CB formation has been an area of significant interest for several decades, and work from many groups has given us an understanding of how early symmetry breaking events culminate in the coordinated rearrangement and sorting of large groups of cells.

## 2. Complementary Domains of Cortical Tension and Junctional Adhesion Drive Cell Intercalation

CE drives elongation of the anterior-posterior body axis in many animal embryos, and it has been studied in the mouse neural plate [9], the *Xenopus* neural tube [10], the *Ciona* notochord [11], the chick neural tube [12], and the *Drosophila* germband [13], among others. The biophysical processes underlying CE have been particularly well characterized in the *Drosophila* germband neuroectoderm, an epithelial tissue comprised of ~3000 cells that doubles in length and halves in width over the course of only 30 min [13] (Figure 1A). At the same time that CE is occurring, the neuroectoderm also becomes simultaneously subdivided into thirteen tissue compartments (also referred to as parasegments) that are separated by CBs [14,15]. The neuroectoderm lies on the surface of the embryo, making it amenable to live imaging studies, and the early phases of CE and compartmentalization occur in the absence of significant tissue growth or division, simplifying analyses of cell morphology. Combined with the power of Drosophila genetics, this tissue has become a paradigm for understanding the molecular bases of cell intercalation and CB formation.

During CE in *Drosophila*, cell-cell contacts between neighbors along the anterior-posterior axis (AP contacts) have relatively high cortical tension and low cell-cell junctional adhesion, whereas contacts between neighbors along the dorsal-ventral axis (DV contacts) have relatively low cortical tension and high cell-cell junctional adhesion [2]. Furthermore, cell-cell contacts at CBs are under even higher tension when compared with non-boundary AP contacts [15]. Cortical tension levels are determined by the activity of non-muscle myosin II (Myo-II) [16–18], a motor protein that can exert force on cell-cell contacts by compacting the network of filamentous actin (F-actin) that underlies the plasma membrane. Cell-cell adhesion is primarily mediated by E-cadherin-based adherens junctions [17,19,20], which are large multiprotein complexes that act like a dynamic glue between the apical domains of adjacent cells. Crucially, myosin activity and junctional stability are affected in opposite ways by the same upstream regulator: Rho kinase (ROK). ROK increases tension by phosphorylating Myo-II [18,21] and inhibits adherens junction stability by phosphorylating the cytoplasmic scaffolding protein Par-3 [19]. ROK is only active at AP contacts because its activator, Rho GTPase, is only active at those contacts [22,23]. Complementary domains of actomyosin-based tension and junction-based adhesion have been observed in a variety of cellular contexts outside of CE [24–26], and appear to be a conserved general mechanism for organizing epithelial cells

## 3. Planar Polarity during *Drosophila* Convergent Extension Requires Patterned Transcriptional Inputs

Before the onset of CE, Myo-II in the neuroectoderm is localized to the cytoplasm, and adherens junctions are homogenously distributed at all cell-cell contacts [17]. The specific activation of ROK leads to the enrichment of active Myo-II and depletion of Par-3 only at AP contacts [16,19] (Figure 1D). This is an example of planar polarity, a phenomenon in which a protein shows a consistent asymmetric pattern of localization or activity across the plane of a tissue, and many proteins involved in CE display planar polarized localization or activity, including ROK, active Rho, F-actin, Myo-II, and Abl at AP contacts, and E-cadherin, Par-3, β-catenin, and α-catenin at DV contacts [2]. However, this raises the question of how neuroectoderm cells can distinguish between cell-cell contacts oriented perpendicular or parallel to the anterior-posterior axis. Two obvious candidates for regulating planar polarity in the neuroectoderm are the core planar cell polarity (PCP) pathway and the Fat/Dachsous pathway, which are highly conserved sets of proteins that organize polarized cell processes in a wide variety of developmental contexts. First described and dissected in the context of *Drosophila* wing hairs [27], the core PCP pathway is also critical for cell polarity in the *Drosophila* compound eye, during mammalian gastrulation and neural tube closure, for polarization of vestibular and auditory cells in the ear, for patterning skin hair follicles, and for certain types of oriented cell division, among others (reviewed in [28,29]). In this system, polarity spreads from cell to cell through interactions between transmembrane proteins, and ubiquitously expressed core PCP proteins become localized to specific cell-cell contacts through local positive and negative feedback loops [29]. However, despite decades of maternal and zygotic screens by numerous labs, to our knowledge, there have been no reported roles for core PCP or Fat/Dachsous components in *Drosophila* CE. Furthermore, gradients of secreted Wnt ligands often impart directionality to core PCPmediated polarity [30]. Interestingly, *wingless*, the *Drosophila* homolog of Wnt, is expressed in a striped pattern during CE [31], making it an attractive candidate for an upstream spatial cue. However, null mutations in *wingless* and the other segment polarity genes have no significant effects on *Drosophila* CE [13]. In this review, we will focus on mechanisms for establishing planar polarity independently of the core PCP and Fat/Dachsous systems during *Drosophila* CE.

**Figure 2.**
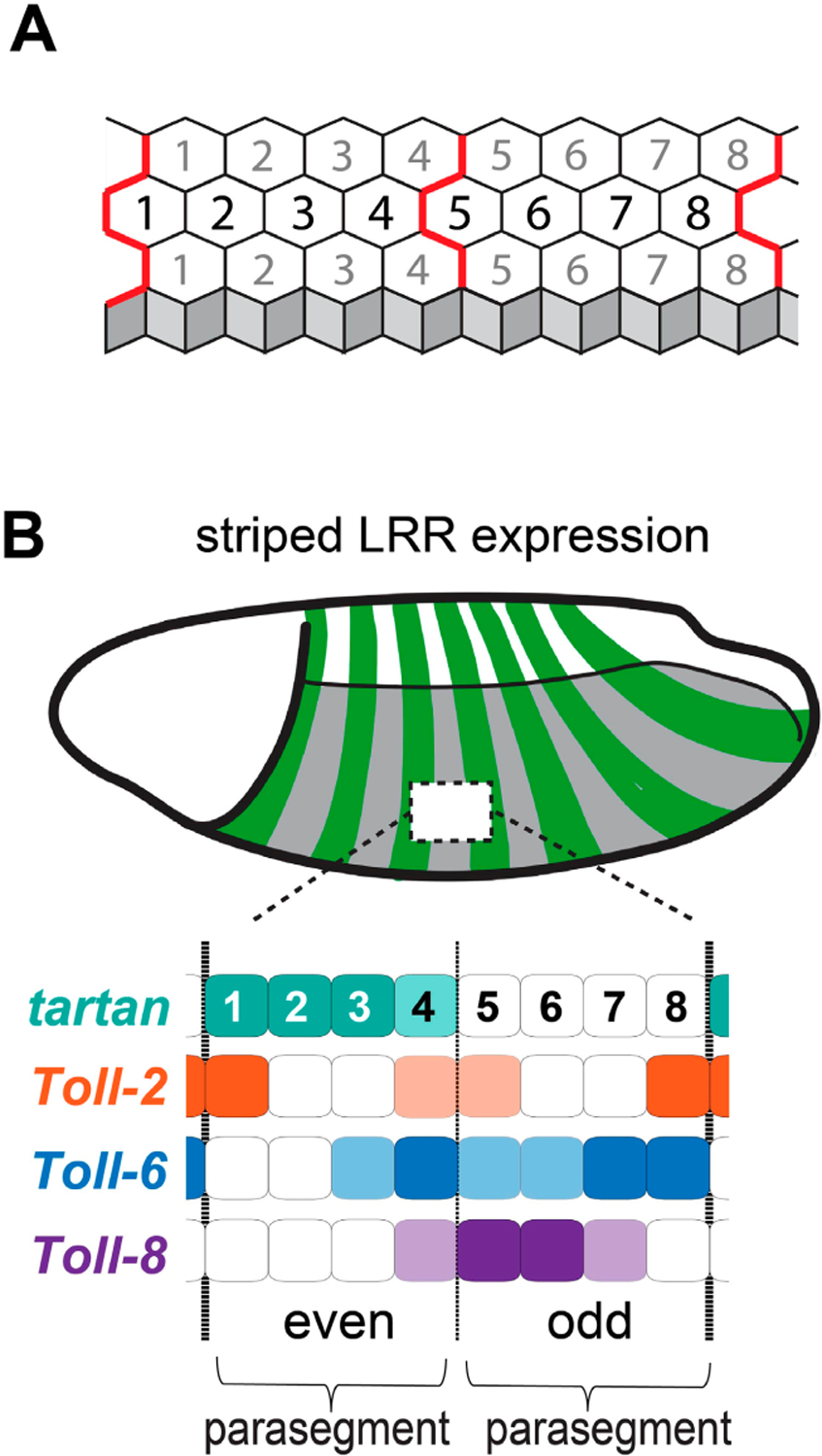

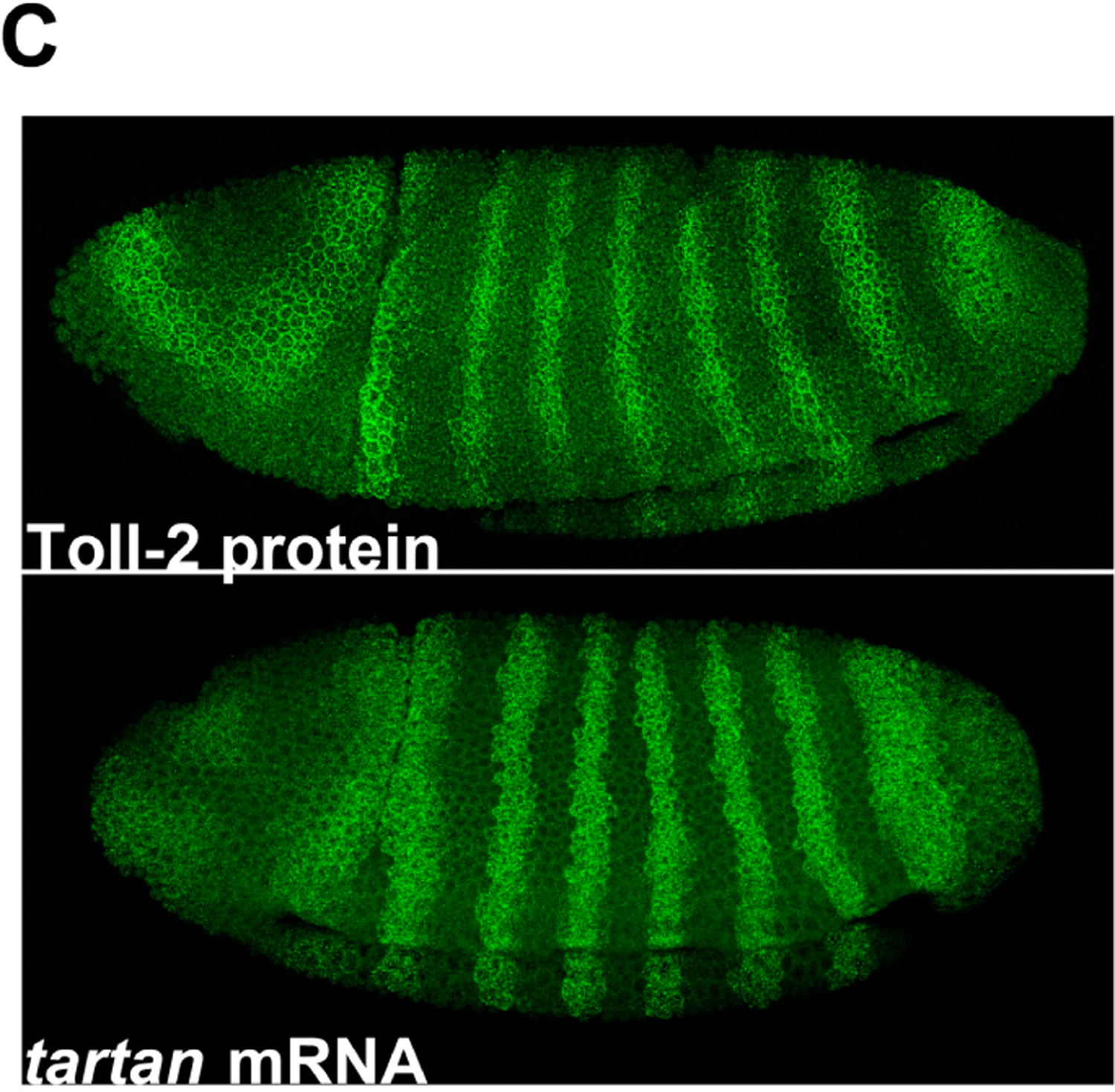
Striped expression of leucine-rich-repeat proteins during convergent extension and compartmentalization. (**A**) Each compartment of the early *Drosophila* embryo consists of two 4-cell-wide parasegments. (**B**) *Toll-2*, *Toll-6*, *Toll-8*, and *tartan* are expressed in 8-column-wide modules that repeat across the anterior-posterior axis. (**C**) Toll-2 protein and *tartan* mRNA are expressed in repeating stripes across the neuroectoderm.

The key mechanistic clue to uncovering the upstream components of planar polarity in the *Drosophila* neuroectoderm was the fact that mutant embryos lacking certain components of the anterior-posterior patterning system have dramatically attenuated CE [13]. These results indicated that the spatial inputs driving CE originate from the anterior-posterior embryonic patterning system, and the pair-rule transcription factors in particular. The anterior-posterior patterning system consists of a set of interacting genes in the early *Drosophila* embryo that (1) creates tandem tissue compartments that will form the basis of the segmented insect body plan, and (2) gives cells distinct identities along the anterior-posterior axis [32]. The original symmetry-breaking event in *Drosophila* embryogenesis is the deposition of specific morphogenetic factors by the mother into the future head and tail of the egg, which through a complex cascade of activating and repressive gene products leads to the non-uniform expression of a class of transcription factors known as the pair-rule genes [33]. Pair rule genes are expressed in regular patterns of seven or eight stripes—roughly four cell columns on and four columns off—across the anterior-posterior axis. There are seven pair-rule genes, each of which has a striped expression profile that is slightly staggered relative to the others, giving each column of cells a distinct complement of pair-rule transcription factors relative to its direct neighbors [34]. These striped patterns of expression repeat every eight columns of cells, and thus the embryo can be thought of as 8-column-wide modules of transcriptional inputs that repeat six times over the length of the neuroectoderm (Figure 2A). Each module corresponds to two parasegments, which are the basic units of cellular organization in the early embryo, and roughly correspond to the future larval and adult body segments [35]. As genes can be activated and repressed in complex ways by multiple pair-rule inputs (as well as other components of the anterior-posterior and dorsal-ventral patterning systems), there is the potential for downstream genes to be expressed in just about any conceivable striped pattern. To serve as spatial cues, it was thought that the pair-rule target genes controlling planar polarity and cell intercalation would (1) be expressed in striped patterns, and (2) encode some type of cell-surface protein or secreted signal that cells could use to communicate information about their orientation within the embryo.

## 4. Striped Leucine-Rich Repeat Receptors Link Embryonic Patterning to Planar Polarity

To identify the pair-rule target genes that control CE, Paré and colleagues knocked down the pair-rule genes *even-skipped* and *runt* using RNAi, and then performed RNAseq analyses on pre-CE embryos [31]. They found that the *Toll-8* and *Toll-2* genes, which encode leucine-rich repeat (LRR) cell-surface receptors, were significantly misexpressed in the knockdown embryos [31]. They confirmed, using fluorescent in situ hybridization, that three members of the Toll family—*Toll-2*, *Toll-6*, and *Toll-8*—are expressed in striped patterns in the *Drosophila* neuroectoderm prior to and during CE (Figure 2B,C) [36–38]. Triple-mutant embryos lacking *Toll-2*, *Toll-6*, and *Toll-8* had strongly defective Myo-II and Par-3 planar polarity, leading to loss of cell intercalation and tissue elongation. In a separate study, it was shown that the loss of individual Toll receptors only affects polarity at specific column borders, and that triple-mutant embryos still display Myo-II and Par-3 polarity at cell-cell contacts that will form the parasegmental CBs, spaced four columns apart [39]. Using a candidate gene approach, they discovered that Tartan—another LRR cell-surface receptor expressed in a striped pattern (Figure 2C) [40]—is required for the polarization of CBs. Critically, they showed that quadruple-mutant embryos lacking *Toll-2*, *Toll-6*, *Toll-8*, and *tartan* lose planar polarity at all cell-cell contacts across the neuroectoderm [39]. Therefore, the striped expression patterns of the LRR receptors *Toll-2*, *Toll-6*, *Toll-8*, and *tartan* are the spatial cues used by *Drosophila* neuroectoderm cells to translate the axial information encoded in the pair-rule genes into planar polarized cell tension and junctional adhesion during CE.

The specific pair-rule genes that control the striped expression patterns of the four LRR receptors have been characterized [41], and the overlapping striped patterns of *Toll-2*, *Toll-6*, *Toll-8*, and *tartan* map well onto the 8-column-wide pair-rule module (Figure 2B). Notably, each cell column in the module expresses distinct combinations and levels of LRR receptors. For example, cell column 1 strongly expresses *tartan* and *Toll-2* but does not express *Toll-6* or *Toll-8*, whereas column 8 strongly expresses *Toll-2* and *Toll-6* but does not express *Tartan* or *Toll-8*. Experiments indicate that it is actually stripe borders—where there is asymmetrical expression across cell-cell contacts—that are critical for triggering planar polarity. For example, in *Toll-2* mutant embryos, Myo-II and Par-3 polarity is strongly decreased at cell-cell contacts between column 1 (Toll-2+) and column 2 (Toll-2−) as well as between column 7 (Toll-2−) and column 8 (Toll-2+); by contrast, polarity at cell-cell contacts between column 8 (Toll-2+) and column 1 (Toll-2+) is not significantly affected [39]. In *tartan* mutant embryos, polarity is strongly decreased at cell-cell contacts between column 4 (Tartan+) and column 5 (Tartan−) as well as between column 8 (Tartan−) and column 1 (Tartan+), whereas polarity is not significantly affected at cell-cell contacts inside the Tartan stripe [39]. There is at least one stripe border at every position of the LRR module (Figure 2B), and evidence suggests that these four receptors account for the vast majority of planar polarity in the *Drosophila* neuroectoderm [39,42,43]. Of course, parasegments are not perfectly rectangular grids of hexagons, and this digital model of receptor expression is a necessary simplification of reality. Notably, there is stochasticity in gene expression that leads to imperfect stripe borders, and parasegments are narrower towards the dorsal side of the embryo, often containing only three columns of cells. Despite these irregularities, the initiation and progression of cell intercalation is remarkably consistent across the tissue [42,44], suggesting that the system is more analog and self-correcting than the digital model might suggest. While the majority of polarity at any given column boundary might be largely controlled by a single receptor, column borders do not become completely depolarized in single-mutant embryos [39], suggesting that the receptors can function in an additive manner. Also, it is known that non-cell-autonomous pulling forces from neighboring cells can enhance Myo-II and adherens junction polarity [45,46], which could be a mechanism for correcting initial stochastic deficiencies in the system.

Structurally, Toll-2, Toll-6, Toll-8, and Tartan are transmembrane proteins expressed at the cell surface that contain arrays of LRRs in their extracellular regions. Tandem LRRs fold into a curved solenoid structure, the interior surface of which consists of a parallel β-sheet, creating a horse-shoe-like architecture that can act as a backbone for local protein-protein and protein-ligand interactions [47]. Vertebrate Toll-like receptors (TLRs) are known to directly bind foreign or pathogenic molecules through their extracellular LRR domains [48], and *Drosophila* Tolls can bind to secreted extracellular protein ligands, such as Spätzle [49]. More generally, LRR proteins can interact with a vast array of other protein types [50], and the direct extracellular interaction partners of the four LRR receptors in the neuroectoderm have not been verified. However, in vivo studies suggest that Toll-8 can interact with the G-protein-coupled receptor (GPCR) Cirl [51] and that Tartan can interact with the teneurin Ten-m [39], although it is not known if these interactions are direct. There is also evidence showing that the extracellular domains of Toll receptors can interact in vitro [31,52], although genetic experiments suggest such interactions may not be relevant during CE in vivo [39,51]. In the remainder of this review, we will discuss in detail what is currently known about how Toll receptors function to control cell intercalation during CE, and how Tartan functions to induce CB formation.

## 5. Toll-2 Signals through Src and PI3K to Induce Cell Intercalation

The Toll receptors are a highly conserved family of single-pass transmembrane proteins found in vertebrates (TLRs) and invertebrates (Toll-related receptors) [53]. Toll family receptors have an extracellular array of LRRs, which can undergo ligand binding, as well as a highly conserved intracellular Toll Interleukin Receptor (TIR) domain, which can mediate both canonical and non-canonical Toll signaling [54]. The founding member of the family, Toll, and its downstream signaling cascade—now known as the canonical Toll signaling pathway—were discovered and characterized in *Drosophila*, where they control patterning of the dorsal-ventral axis of the embryo [55–57]. Later, it was found that vertebrate TLRs are pathogen recognition proteins and that canonical Toll signaling pathways are required for the innate immune response in most animals [58]. Over the last three decades, many thousands of papers have been published on the role of Toll family receptors in the context of innate immunity and inflammation, and the 2011 Nobel Prize was awarded for pioneering work in this area. By contrast, direct functions for Toll receptors in regulating cell shape and polarity have received significantly less attention, although numerous studies indicate that Toll family receptors are important for epithelial morphology [5,36–38,52,59–64], nervous system development [65–69], wound healing [70,71], and cell competition [72].

In search of the pathways linking Toll receptors to planar polarity during CE, Tamada and colleagues in the Zallen group hypothesized that tyrosine phosphorylation could be playing a role in receptor signaling [73]. They observed that an antibody specific to the active phosphorylated form of the tyrosine kinase Src42 is enriched at AP contacts and that this polarity is lost in *Toll*-*2*, −*6*, and −*8* triple-mutant embryos [73]. Simultaneous knockdown of Src42 and Src64 activity in the early embryo led to a dramatic decrease in the polarity of Myo-II and Par-3, similar to Toll triple-mutant embryos, whereas ubiquitous overexpression of a constitutively active form of Src42 caused Myo-II to be recruited to both AP and DV contacts [73]. Using a systematic mutational approach and co-immunoprecipitation experiments, they showed that phosphorylated tyrosine residues in the C-terminal region of Toll-2 enhance the physical association between Toll-2 and Src42, whereas tyrosine residues in the TIR domain are dispensable for this association [73]. Considering that PI3K can be recruited to phosphorylated receptors in other cellular contexts [74], Tamada and colleagues next investigated whether PI3K is downstream of Toll-2 and Src during CE. They found that the regulatory subunit of the PI3K complex is enriched at AP contacts in a Toll-dependent manner and that CE is significantly reduced in PI3K-knockdown embryos [73]. Inhibition of Src42 also disrupted PI3K localization and function, and they showed in vivo that the C-terminal tyrosine clusters in Toll-2 are required for the polarity of Myo-II, Par-3, and PI3K at the borders of Toll-2 stripes [73].

**Figure 3.**
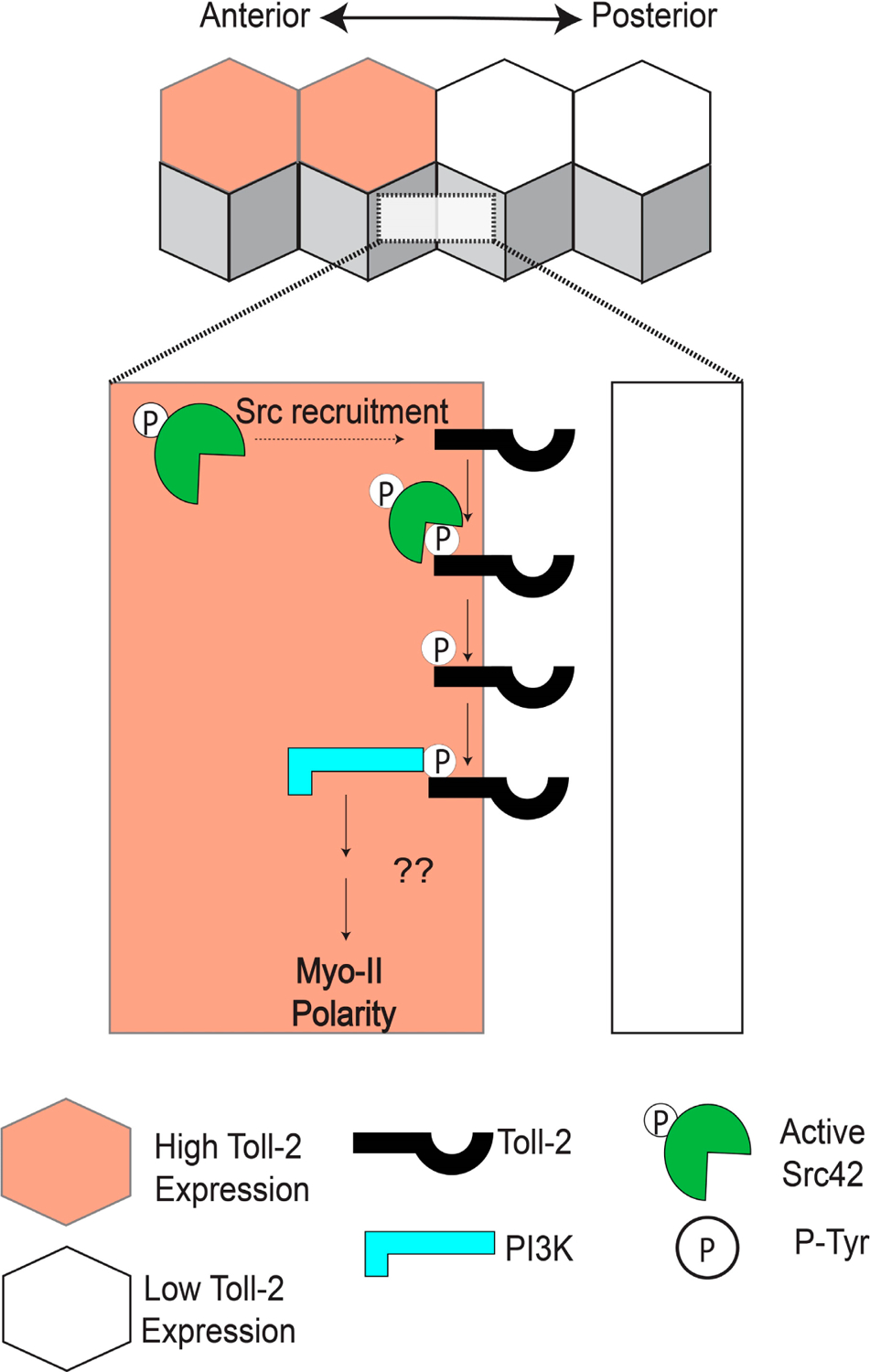
Toll-2 acts upstream of Src and PI3K during convergent extension. Toll-2 is phosphorylated by Src at cell-cell contacts between neighboring cells expressing different levels of Toll-2. Phosphorylation of the C-terminal region of Toll-2 by Src is required to recruit PI3K, which induces Myo-II polarity.

Combined, these results suggest a model in which active Src42 is recruited to and phosphorylates Toll-2 at AP contacts. The PI3K complex would then bind to the phosphorylated C-terminal tyrosine residues of Toll-2, leading to the polarization of Myo-II and Par-3 along the borders of Toll-2 stripes (Figure 3) [73]. Such a mechanism would be consistent with the rapid establishment of polarity and induction of cell intercalation following the striped expression of Toll receptors. However, several outstanding questions remain. Most notably, it is unknown why active Src is only enriched at AP contacts, and not at DV contacts. This phenomenon is presumably linked to the striped expression patterns of the Toll receptors, although non-uniform expression alone is not a sufficient explanation, considering DV contacts also have Toll receptors at the membrane [31]. Therefore, there must be a mechanism that biases Src-dependent Toll-2 phosphorylation toward AP contacts that involves either the planar polarized activation or localization of the Toll receptors themselves. Furthermore, the direct molecular links between PI3K, Myo-II, and Par-3 at AP contacts remain unidentified. One compelling hypothesis is that PI3K alters the membrane composition of AP contacts through the production of phosphatidylinositol (3,4,5)- trisphosphate (PIP3), eventually culminating in polarized ROK activity. Rho-GEFs can be recruited to PIP3-enriched membranes through their pleckstrin homology (PH) domains [75,76], leading to more active Rho and, in turn, more active ROK, which could then directly phosphorylate Myo-II and Par-3 [18,19,21]. Mutational studies have yet to identify a single Rho-GEF that has effects on CE comparable to loss of the Toll receptors or Src, but evidence suggests that multiple Rho-GEFs may act redundantly to activate Rho [23]. Further studies will be required to establish a direct link between PI3K signaling and ROK during CE.

## 6. Toll-8 Interacts with the GPCR Cirl to Establish Planar Polarity

Work by the Lecuit group has established that GPCR signaling in the *Drosophila* neuroectoderm is necessary for Rho and Myo-II activity at adherens junctions [23,77]. To investigate potential links between GPCR signaling and Toll-mediated planar polarity during CE, Lavalou and colleagues used co-immunoprecipitation and mass spectrometry experiments to identify the adhesion GPCR Cirl—the *Drosophila* ortholog of the vertebrate latrophilin—-as the most abundant Toll-8 interaction partner [51]. An overexpressed tagged version of Cirl localized to the cell membrane around adherens junctions in the neuroectoderm, and *Cirl* null-mutant embryos, showed decreased tissue elongation and lower total levels of Myo-II at both AP and DV contacts [51]. To investigate interactions between Toll-8 and Cirl, they next used clonal analyses in the wing disc to examine groups of cells expressing one, both, or neither of the receptors. They found that both Myo-II and Cirl were enriched at the borders of Toll-8+ and Toll-8− cell groups, forming clones with smooth borders [51]. Unexpectedly, they also showed that Myo-II polarity was induced at clonal borders between Cirl+ and Cirl− cells, independent of whether Toll-8 was present [51]. To better characterize the dynamics of Toll/Cirl interactions, Lavalou and colleagues performed live imaging to track changes in the localization of Toll-8 and Cirl during clone growth. Interestingly, they found that Cirl was enriched not only at borders between Toll-8+ and Toll-8− cells, but also between cells with low vs. high levels of Toll-8, indicating that Cirl is sensitive to quantitative differences in Toll-8 levels [51]. Furthermore, they observed that Toll-8 itself was often planar polarized, becoming enriched at clone borders and depleted from orthogonal contacts, and they showed that Toll-8 planar polarity disappears in a *Cirl* mutant background [51]. Finally, they noted that Cirl had a differential localization along the apical-basal axis in the wing disc that was dependent on the juxtaposition of neighboring cells with different Toll-8 expression levels [51].

**Figure 4.**
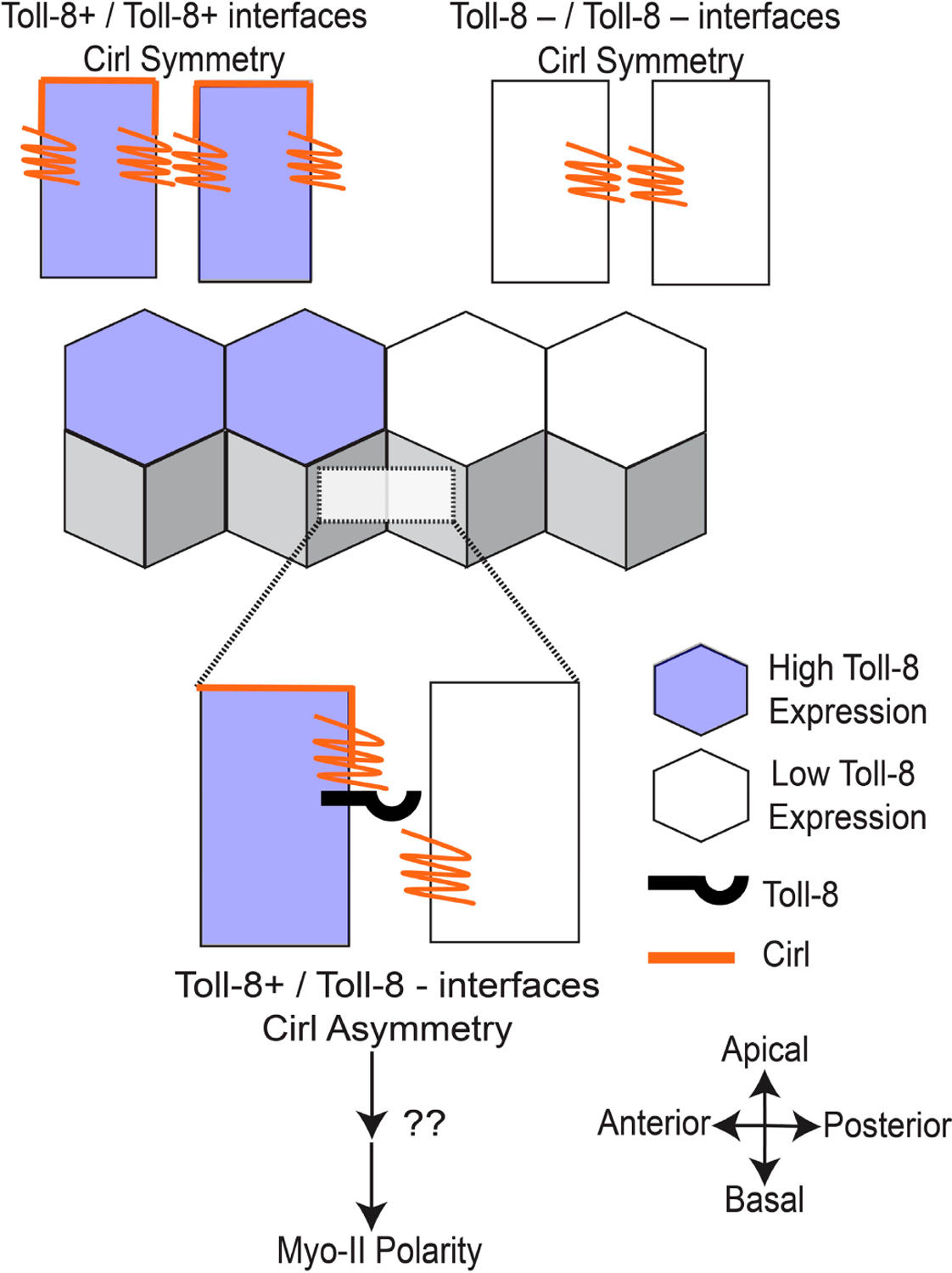
Toll-8 generates asymmetric Cirl localization upstream of Myo-II polarity. The juxtaposition of Toll-8 expression between neighboring cells results in Cirl asymmetry along the apical-basal axis. The asymmetric distribution of Cirl is sufficient to induce Myo-II polarity at contacts along the Toll-8 expression border.

Considering that Toll-8 is required for Cirl polarity, and Cirl is required for Toll-8 polarity, the researchers proposed a model in which initial asymmetries in Toll-8 expression levels between neighboring cells are amplified by physical *trans* interactions between Toll-8 and Cirl, which would lead to the enrichment of both proteins at clone borders and their depletion from orthogonal contacts; then, the juxtaposition of Toll-8+ and Toll-8− cells would lead to asymmetries in the apical-basal localization of Cirl around adherens junctions, which they proposed is the direct signal for downstream activation of Myo-II (Figure 4) [51]. Consistent with this model, they showed that the misexpression of Toll-8 in a horizontal stripe in the neuroectoderm is sufficient to induce Myo-II polarity at the ventral stripe border, and critically they showed that this ectopic Myo-II polarity is Cirl-dependent [51]. To date, the signaling pathways downstream of Cirl that trigger Myo-II polarity have not been revealed. It is interesting to note that, in the wing disc, Myo-II was enriched on both sides of Toll-8 clone borders, even in cells lacking endogenous Cirl [51]. This suggests that there are bidirectional signaling pathways downstream of asymmetrical Toll-8 that can polarize Myo-II in both Cirl+ and Cirl− cells, or that biomechanical feedback loops are sufficient to propagate myosin polarity from one side of a cell-cell contact to the adjacent cell [45]. Furthermore, Lavlou and colleagues did not report seeing a planar polarized distribution of their transgenic tagged versions of Cirl or Toll-8 in the neuroectoderm during CE. Considering differences in timescale and cell type, it is possible that the same Toll-8/Cirl interactions are occurring in the wing disc and the neuroectoderm, with the exception that Toll-8 and Cirl do not become significantly polarized in the latter tissue. Alternatively, planar polarized localization or activity patterns for the endogenous Cirl and Toll-8 proteins might be detectable using other methods. Finally, it is interesting to note that, in the wing disc, Cirl can become polarized in response to quantitative, not just qualitative, differences in Toll-8 expression levels [51]. During CE, the Toll-2 stripes have very sharp borders, whereas the Toll-8 stripes are more graded and sinusoidal [39], and we speculate that these different expression patterns may have influenced the evolution of their downstream polarization mechanisms, or vice versa.

## 7. Compartment Boundaries in the Early *Drosophila* Embryo Require Tartan and Ten-m

A significant challenge faced by developing organisms is how to keep distinct epithelial cell populations from mixing together. To counteract cell mixing, CBs are formed between distinct pools of cells by aligning cell-cell contacts into highly stable, actomyosin-rich structures that resist cell movements between compartments [6–8,78–80]. CBs were first discovered in insects [81–83], and they have since been described in many vertebrate tissues, notably between different regions of the brain [84–88], between somites [89], within limb buds [90–93], and in the gut [94]. Furthermore, evidence suggests that the disruption of CBs can contribute to cancer metastasis [95,96] and birth defects such as craniofrontonasal syndrome [97]. *Drosophila* CE is an excellent model in which to study CB formation, as the neuroectoderm not only elongates but also becomes divided into 13 parasegment compartments, each separated by a CB (Figure 1A). As with non-boundary AP contacts, nascent CBs are characterized by enriched Myo-II and depleted Par-3, although Myo-II levels and tension are significantly higher at CBs when compared with non-boundary contacts [15,45,98]. Importantly, CB contacts do not undergo intercalation per se, but rather remodel their junctions to align AP contacts (Figure 1C) [8]. Aside from their biomechanical properties, little is known about what makes CBs molecularly distinct from AP contacts in the neuroectoderm that allow them to serve as long-term barriers to cell mixing. However, the upstream mechanisms that position and maintain CBs in the *Drosophila* embryo have now been identified.

**Figure 5.**
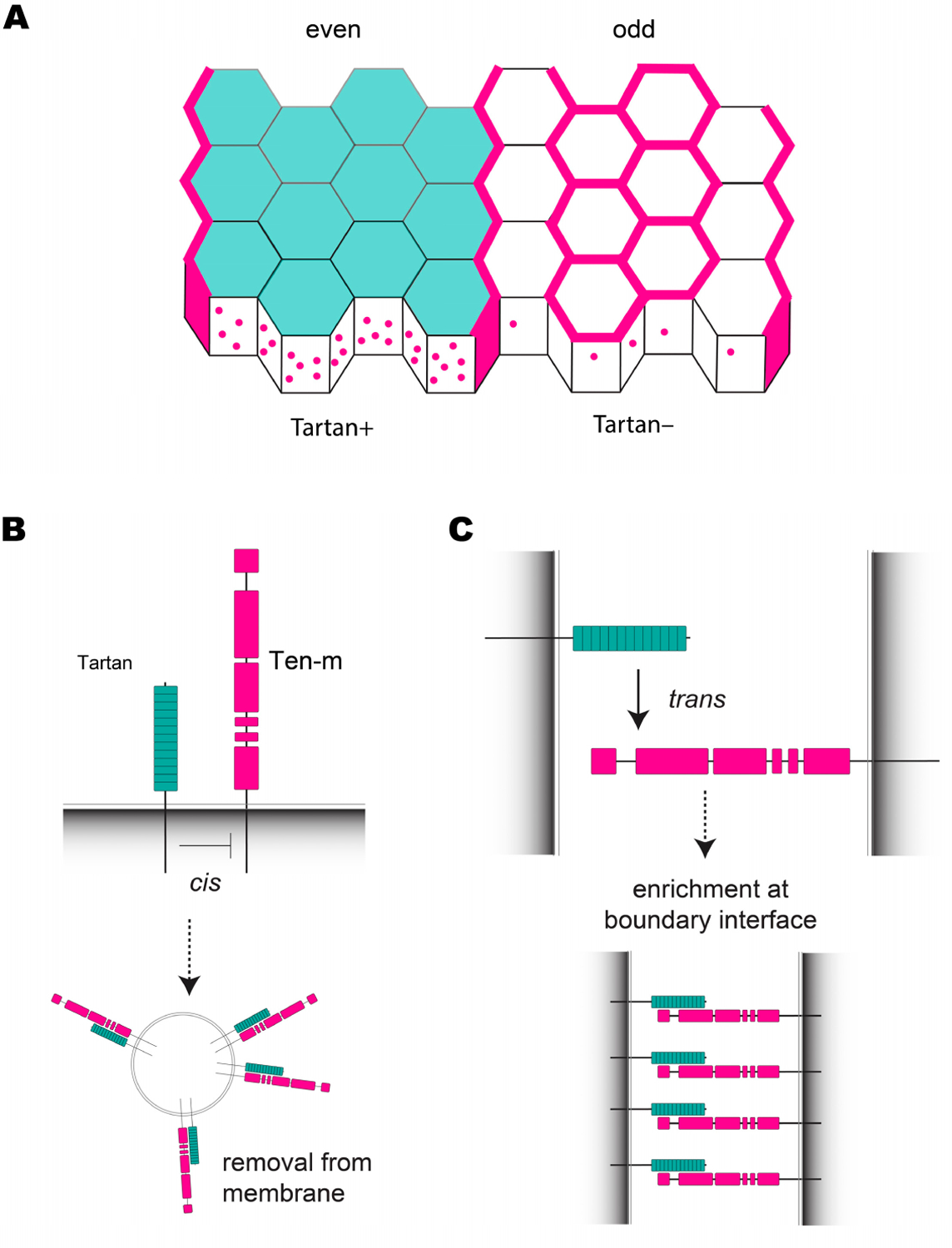
Tartan and Ten-m are required to establish planar polarity at CBs. (**A**) Alternating stripes of *tartan* (teal) expression coincide with even-numbered parasegments. Ten-m (pink) is not present at the membrane in Tartan+ cells, but localizes to the membrane in Tartan− cells. Ten-m is strongly enriched at CBs between Tartan+ and Tartan− cells. (**B**) A model for Ten-m removal from the cell membrane by endocytosis in Tartan+ cells through *cis* interactions. (**C**) A model for Ten-m membrane enrichment at CBs mediated by *trans* interactions between Tartan+ and Tartan− cells8. Tartan Interacts with Ten-m to Initiate Compartment Boundary Formation.

Wingless—which is also expressed in a striped pattern downstream of the pair-rule genes—is necessary for maintaining parasegmental boundaries in *Drosophila* and arthro-pods in general, although CBs still initially form correctly in *wingless* mutant *Drosophila* embryos [15,43,98]. Furthermore, embryos lacking *Toll-2*, *−6*, and *−8* expression still display Myo-II and Par-3 planar polarity at CBs [39], indicating that Toll receptors do not control boundary formation. Assuming planar polarity is triggered along the borders of receptor stripes, a parsimony model predicted that an additional polarity cue (aside from Toll-2,−6, and −8) expressed in alternating parasegments was required to account for planar polarity at all cell column borders across the neuroectoderm [42]. Consistent with this hypothesis, the LRR receptor Tartan is expressed in a striped pattern and is present at the cell membrane in even-numbered parasegments (Figures 2B and 5A) [39,40]. In *tartan* mutant embryos, Myo-II polarity and CB straightness are both strongly disrupted along the missing Tartan stripe borders, whereas non-boundary contacts are unaffected [39]. Thus, Tartan was identified as a fourth LRR receptor that mediates polarity during CE, independent of the Tolls. In the same study, it was shown that Teneurin-m (Ten-m) is enriched at CBs at the onset of CE, and like Tartan, Ten-m knockdown also disrupted polarity and boundary straightness specifically at CBs but not at other AP contacts [39]. These findings suggested a mechanism in which CBs are positioned by the striped expression of Tartan, with Ten-m acting downstream of Tartan to induce planar polarity.

Immunohistochemistry experiments indicate that the Ten-m protein is ubiquitously expressed in all neuroectoderm cells, but it is strongly enriched at the cell membrane along the borders of Tartan stripes, where Tartan+ and Tartan− cells come into contact [39]. In Tartan-expressing cells (columns 1–4), Ten-m is absent from the membrane at DV contacts and at non-boundary AP contacts, although it is present in numerous puncta of concentrated protein signal within the cytoplasm that colocalize with Tartan. Interestingly, Ten-m is present at both AP and DV contacts in cell columns 6 and 7, which do not express Tartan or contact Tartan-expressing cells, and it is depleted from DV contacts in columns 5 and 8, likely through relocalization to CBs (Figure 5A). This striking Ten-m localization pattern is completely dependent on the striped expression of Tartan. In *tartan* mutant embryos, Ten-m is present at the cell membrane in all cells, whereas in embryos that ubiquitously overexpressed Tartan, Ten-m was only found in cytoplasmic puncta [39]. A model was suggested in which (1) a *cis* interaction between Tartan and Ten-m in the same cell cause Ten-m to be removed from the membrane via endocytosis (Figure 5B), and (2) a *trans* interaction between the extracellular domains of Tartan and Ten-m on neighboring cells can override the *cis* interaction, allowing Ten-m to remain at the membrane along the borders of Tartan stripes (Figure 5C). Consistent with this model, endogenous Ten-m protein was recruited to sites of contact between Tartan+ and Tartan− cells in cultured S2 cells [39], suggesting these proteins can physically interact in *trans*. The Tartan/Ten-m system is an example of how two interacting cell-surface proteins—one with patterned expression and one without—can induce planar polarity, reminiscent of Toll-8 altering the localization of Cirl [51], Notch ligands altering the localization of Notch [99], and the polarization of core PCP components in response to Fat gradients [100].

It is not currently known how Tartan and Ten-m signal intracellularly to induce planar polarity. Tartan and the closely related protein Capricious have been studied in other *Drosophila* tissues, where they regulate cell-cell affinity in the morphogenetic furrow of the eye disc [101], at the dorsal-ventral CB in the wing disc [102], at the tarsal/pretarsal CB in the leg disc [103], and at branch connections during tracheal tube formation [104]. The intracellular region of Tartan is not well conserved, even among insects, and it appears necessary for some of the above processes while dispensable for others, so it is possible that Tartan may strictly serve as a ligand for Ten-m during *Drosophila* CB formation. Ten-m is a member of the highly conserved teneurin family of type II cell-surface proteins, which have an extensive extracellular region containing numerous protein-protein interaction domains [105], and there is evidence that Tenm3 can interact with the LRR protein LRRTM2 in mammals [106]. Teneurins act as cell-adhesion molecules during synapse formation in *Drosophila* [107–110] and vertebrates [111–114], and groups of interconnected neurons often express the same teneurin type [105]. It is possible that one of the functions of Ten-m is to act as a cell-adhesion molecule to stabilize cell-cell contacts at CBs, and indeed endogenous Ten-m protein extends quite far basally at CBs [39]. Intracellularly, teneurins contain SH3 binding domains that have been linked to cytoskeletal organization in other systems [115], and *Drosophila* Ten-m was shown to regulate *α*-spectrin organization in the neuromuscular synapse [108]. Therefore, it seems likely that Ten-m has direct effects on cytoskeletal organization at CBs, and future studies involving super-resolution microscopy or cell-type-specific biochemistry could address the molecular nature of such interactions.

A recent study revealed that the role of Tartan at CBs is surprisingly transient, and it is only required for Myo-II polarity and CB straightness during CE (stages 7 and 8), but not during subsequent extended stages [43]. CB straightness recovers during stage 9 in *tartan* mutant embryos, and this later polarization is dependent on Wingless signaling [43]. Mechanistically, it is still unknown how Wingless signaling enhances CB straightness and tension, and it is possible that the presence of Ten-m primes CBs for later Wingless-mediated processes. Furthermore, it is an open question as to whether Ten-m protein is present on both sides of CB contacts during *Drosophila* CE, and it could be that homotypic *trans* interactions also stabilize Ten-m in Tartan+ cells. Alternatively, it is possible that CBs are asymmetric with respect to Ten-m, which could allow for unidirectional signaling, perhaps involving secreted Wingless ligand across the boundary. It is currently unknown whether there are interactions between Toll receptors and Tartan, but it is interesting to note that while they mediate distinct cell behaviors (intercalation vs. CB formation), they share at least two critical downstream effectors (Myo-II and Par-3). Toll-2 stripes also straddle CBs during later stages of development [36,37], and this expression pattern is partly dependent on Wingless signaling [43], although there is currently no evidence that Toll-2 directly affects CB maintenance. Interestingly, it was recently reported that Toll-1 is expressed on one side of histoblast CBs in pupae, where it appears to regulate boundary straightness through cell-cell adhesion [52]. Therefore, future studies should address how Tartan/Ten-m, Wingless, and perhaps Toll receptors interact to form and maintain CBs during development.

## 8. Functional Overlap between LRR Receptor Signaling Pathways during CE

It was hypothesized that heterotypic *trans* interactions between the extracellular regions of different Toll receptor types are necessary for biasing receptor activation towards AP contacts [31]. This idea was supported by cell culture experiments indicating that S2 cells expressing one type of receptor more often bound to S2 cells expressing a different receptor type than they did to untransfected cells [31]. However, multiple Toll receptor types can induce S2 cell clumping when expressed individually [31,52,116], and it is not clear how these results relate to epithelial cell behavior. In addition, it was demonstrated that a pentamerized version of the Toll-2 extracellular region strongly bound to Toll-8-expressing cells but not to untransfected cells [31,50]. However, this experiment did not discriminate between *trans* vs. *cis* orientations for the proposed Toll-2/Toll-8 interaction, and it is well established that different Toll family proteins can form heterodimers when present in the same cell [117,118]. Furthermore, considering that one neuroectoderm cell can express multiple Toll receptor types (Figure 2B), it is unclear why heterotypic interactions within cell columns would not also recruit Myo-II to DV contacts. Compelling genetic evidence also argues that heterotypic interactions are not required for Toll receptors to initiate planar polarity. For example, if one supposes that heterotypic interactions between Toll-2 and Toll-8 are strictly required and activate both receptor types bidirectionally, then you would predict that a single mutant lacking *Toll-2* would be just as defective as a double mutant lacking both *Toll-2* and *Toll-8*. However, this is not the case, as single mutants display localized defects in planar polarity at distinct column borders, and planar polarity is significantly lower in double-mutant backgrounds when compared with single mutants [31,39]. Furthermore, the expression of an ectopic horizontal stripe of Toll-8 across the neuroectoderm was sufficient to induce Myo-II polarity, which occurred even when the endogenous *Toll-2*, *Toll-6*, and *Toll-8* genes were knocked down [51]. These results indicate that heterotypic interactions are likely not important for the function of Toll receptors during CE, and the preponderance of evidence is more consistent with models in which each Toll receptor type can trigger polarity independently of the others [2].

As discussed in this review, recent studies have begun to identify the signaling pathways downstream of the LRR receptors during CE. Notably, it was shown that Src and PI3K signaling are activated downstream of Toll-2 [73], and an interaction between Toll-8 and Cirl appears necessary for Toll-8 activity [51]. It is somewhat puzzling why two completely different signaling systems would have evolved downstream of such closely related receptors, especially considering the ultimate cell biological outputs—polarized tension and junctional adhesion leading to cell intercalation—are the same. But evidence suggests that there may in fact be significant overlap between the Src/PI3K and GPCR signaling pathways downstream of all three receptors. For example, Toll-2 and Toll-6 were both phosphorylated on C-terminal tyrosine residues by Src in cultured cells, although Toll-8 was not; however, Toll-8 could be immunoprecipitated with PI3K in cultured cells, and simultaneous knockdown of both Toll-2 and Toll-8 was necessary for strong negative effects on PI3K polarity [73]. Future studies should systematically address the contributions of *Toll-2*, *Toll-6*, and *Toll-8* mutants toward Src and PI3K activity. Lavalou and colleagues also noticed intriguing similarities and differences between the functions of Toll-2, Toll-6, and Toll-8 in the wing disc. Notably, all three Toll receptors could induce Myo-II polarity at clone borders, which did not require the cytoplasmic regions of these receptors [51], suggesting that these effects do not require intracellular Src phosphorylation; by contrast, Toll-8 and Toll-6 could both induce Cirl polarity in wing discs, whereas Toll-2 could not [51]. However, the relevance of these wing-disc results to CE remains to be addressed. An interesting possibility is that Toll-2 has an unidentified interaction partner that functions analogously to Cirl and is required for the activation of Toll-2 specifically at AP contacts during CE. Finally, Src knockdown seems to have very strong effects on polarity across the neuroectoderm [73], suggesting that Src may also be important for polarization at CBs. Consistent with this, there is evidence that Tartan can be phosphorylated by Src in S2 cells [119], although potential interactions between Tartan, Ten-m, Src, and PI3K have not been studied during CE.

## 9. Concluding Remarks

Planar polarity is a common phenomenon of symmetry breaking observed during vertebrate and invertebrate development that allows for the existence of polarized cellular processes across the plane of a tissue. The LRR receptor-mediated polarity mechanisms discussed in this review appear to function independently of the better-known core PCP and Fat/Dachsous pathways, making them exciting new paradigms for understanding how cytoskeletal and junctional proteins can polarize in response to non-uniform receptor expression. However, many fundamental questions remain regarding the molecular function of these receptors during cell intercalation and CB formation, as well as the wider importance of LRR receptor-mediated planar polarity across animals. Notably, it is unknown what molecules Toll-2 and Toll-6 are interacting with extracellularly to become activated in a planar polarized fashion; and for Toll-8 and Cirl, it is unclear how relevant the mechanisms observed in imaginal discs are for CE. It will also be interesting to better characterize the similarities and differences between the signaling pathways downstream of the Toll receptors, which could shed light on why the LRR module is laid out the way it is, and how these receptors might function in other contexts. Furthermore, quadruple null mutants for *Toll-2*, *Toll-6*, *Toll-8*, and *tartan* still show residual planar polarity around cell columns 4 and 5 in the neuroectoderm [39], indicating there are polarity cues yet to identify in this system. With respect to CBs, super-resolution microscopy or cell-type-specific biochemical methods should be used to determine whether Ten-m is present only at the anterior, only the posterior, or on both sides of the CB, and also whether Myo-II shows asymmetries in abundance or organization across CBs. Outside the neuroectoderm, Toll receptors and Tartan are expressed in striking non-uniform patterns in the late embryo and in imaginal discs, and as discussed above, they have been implicated in numerous polarized cellular processes besides cell intercalation and CB formation. For example, *Drosophila* Toll receptors have been linked to epithelial remodeling [5,36–38,52,59–64], nervous system development [65–69], wound healing [70,71], and cell competition [72], and the lessons learned during CE could shed new light on the molecular nature of these processes. Finally, TLRs and other LRR receptor proteins are widely expressed throughout vertebrate tissues [64,68,120,121], and they also play well-known roles in immunity, inflammation, and wound healing. While vertebrate researchers may never find striped LRR receptor expression modules akin to that in the *Drosophila* neuroectoderm, there may only be the need for discontinuities in receptor expression levels between neighboring cells to achieve some effect on cell morphology or function. Therefore, it will be very interesting to discover whether non-uniform LRR receptor expression is a general conserved mechanism for controlling epithelial and cell morphology during vertebrate development and adult homeostasis.

## Data Availability

No new data were generated in the course of writing this review article.
